# Immunization of mice with the *nef *gene from Human Immunodeficiency Virus type 1: Study of immunological memory and long-term toxicology

**DOI:** 10.1186/1750-9378-2-14

**Published:** 2007-07-10

**Authors:** Andreas Bråve, Lindvi Gudmundsdotter, Georg Gasteiger, Kristian Hallermalm, Wolfgang Kastenmuller, Erik Rollman, Andreas Boberg, Gunnel Engström, Sven Reiland, Antonio Cosma, Ingo Drexler, Jorma Hinkula, Britta Wahren, Volker Erfle

**Affiliations:** 1 Swedish Institute for Infectious Disease Control, 17182 Solna, Sweden; 2 Department of Microbiology, Tumor and Cell Biology, Karolinska Institute, 17177 Stockholm, Sweden; 3 Institute of Molecular Virology, GSF-National Research Center for Environment and Health, Ingolstaedter Landstrasse 1a, 85764 Neuherberg, Germany; 4 Institute for Virology at Technical University of Munich, Trogerstr. 4b, D-81675 München, Germany; 5 Department of Microbiology and Immunology, University of Melbourne, Royal Parade, Vic. 3010, Australia; 6 Biovet AB, Box 1013, 19221 Sollentuna, Sweden

## Abstract

**Background:**

The human immunodeficiency virus type 1 (HIV-1) regulatory protein, Nef, is an attractive vaccine target because it is involved in viral pathogenesis, is expressed early in the viral life cycle and harbors many T and B cell epitopes. Several clinical trials include gene-based vaccines encoding this protein. However, Nef has been shown to transform certain cell types *in vitro*. Based on these findings we performed a long-term toxicity and immunogenicity study of Nef, encoded either by Modified Vaccinia virus Ankara or by plasmid DNA. BALB/c mice were primed twice with either DNA or MVA encoding Nef and received a homologous or heterologous boost ten months later. In the meantime, the Nef-specific immune responses were monitored and at the time of sacrifice an extensive toxicological evaluation was performed, where presence of tumors and other pathological changes were assessed.

**Results:**

The toxicological evaluation showed that immunization with MVAnef is safe and does not cause cellular transformation or other toxicity in somatic organs.

Both DNAnef and MVAnef immunized animals developed potent Nef-specific cellular responses that declined to undetectable levels over time, and could readily be boosted after almost one year. This is of particular interest since it shows that plasmid DNA vaccine can also be used as a potent late booster of primed immune responses. We observed qualitative differences between the T cell responses induced by the two different vectors: DNA-encoded nef induced long-lasting CD8^+ ^T cell memory responses, whereas MVA-encoded nef induced CD4^+ ^T cell memory responses. In terms of the humoral immune responses, we show that two injections of MVAnef induce significant anti-Nef titers, while repeated injections of DNAnef do not. A single boost with MVAnef could enhance the antibody response following DNAnef prime to the same level as that observed in animals immunized repeatedly with MVAnef. We also demonstrate the possibility to boost HIV-1 Nef-specific immune responses using the MVAnef construct despite the presence of potent anti-vector immunity.

**Conclusion:**

This study shows that the nef gene vectored by MVA does not induce malignancies or other adverse effects in mice. Further, we show that when the nef gene is delivered by plasmid or by a viral vector, it elicits potent and long-lasting immune responses and that these responses can be directed towards a CD4^+ ^or a CD8^+ ^T cell response depending on the choice of vector.

## Background

In the challenging race for an HIV-1 vaccine, many researchers are considering genetic vaccines, either based on viral vectors or as naked DNA vaccines, due to their ability to induce protective immune responses in animal models [1]. Although both modalities induce potent cellular and humoral responses in animals [2,3] there is a need to improve the potency of these types of vaccine [4]. To improve and broaden immune responses against HIV-1, combinations of viral genes encoding structural, enzymatic and regulatory proteins of HIV-1 have been used [5-8]. The HIV-1 regulatory genes tat and nef are attractive as vaccine components since both are immunogenic and harbor several T and B cell epitopes. In addition, they are expressed early in the viral life cycle and are relatively well conserved. These features have led to several vaccine trials using the regulatory proteins, trials in which the proteins have been concluded immunogenic and safe in both humans and animals [9-12]. However, the regulatory genes of HIV-1 have been shown to influence cellular functions and also to interact with several mechanisms of the host immune response. Nef has been shown to be important for viral replication, viral pathogenesis and progression to AIDS in infected persons [13,14]. The protein exhibits many intricate actions for helping the virus avoid the immune system, including a potent down-regulation of surface markers such as MHC class I and CD4 molecules (reviewed in [15]). More recently, Nef was shown to redistribute the co-stimulatory molecules CD80 and CD86 away from the cell surface of antigen presenting cells [16]. Furthermore, Nef is able to interact with the SH3 domain of Hck, a tyrosine kinase of the Src family. This interaction can lead to the transformation of fibroblasts and neuronal cells *in vitro *[17-20]. In addition, experiments using transgenic mice constitutively expressing nef in cells of the kidney support the protein's contribution to the development of HIV-1 associated nephropathy (reviewed in [21]). However, little is known about the long-term effects after immunization with Nef. Transgenic mice are well-suited for studying the role of Nef during HIV-1-infection, since the protein in these models is continuously expressed in the animals. They are, however, inappropriate for studying the effects after genetic immunization, in which the protein is expressed only transiently. To assess the potential long-term toxicity following nef-immunization in a model that allows for full necropsy and extensive investigation of the major organs, we immunized mice with a recombinant viral vector, MVA, encoding Nef. This construct has been evaluated for safety and immunogenicity in a clinical phase I trial [10]. We chose to use BALB/c mice for evaluation of the long-term toxicity and tumorgenicity of the vaccine construct, since this strain of mice is less prone to spontaneous tumor development than other inbred mice [22-24]. Thus, the relative stability of BALB/c mice might better mimic the conditions in an outbred human population. The normal life span of female BALB/c mice is approximately twenty-four months [25]. By evaluating the toxicity of the nef-encoding MVA construct in this mouse strain over one year, we believe we have a good model for assessing possible long-term side effects induced by Nef-vaccination.

In parallel with the long-term toxicology, we explored the long-term immune responses elicited either by repeated single modality immunization or by heterologous plasmid DNA prime MVA boost regimen.

## Results

### Toxicology and histopathology

**Figure 1:**
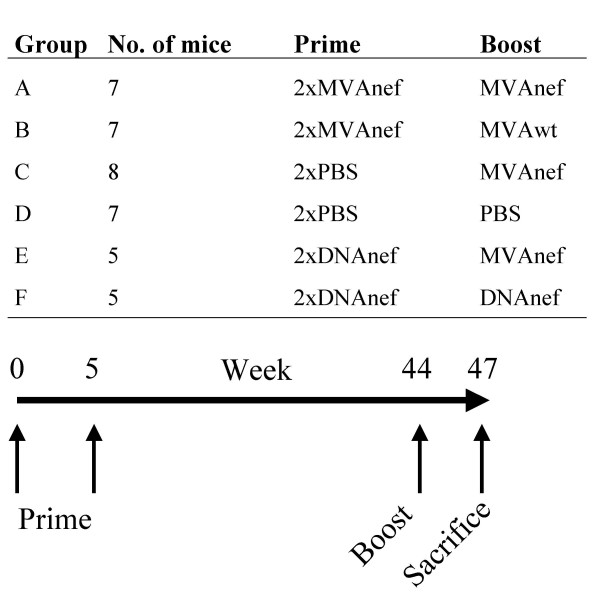
Experimental setup.

We immunized BALB/c mice with two injections of either DNAnef intramuscularly or MVAnef intraperitoneally. Forty weeks later the animals received a homologous or heterologous boost (see Figure 1 for immunization protocol). Three weeks after the late boost animals were sacrificed and the mice immunized with MVAnef were subjected to extensive toxicological investigations, performed according to good laboratory practice (GLP).

#### Gross pathology

The immunization schedule included two injections at weeks 0 and 5 and a boost at week 44 (Figure 1). Necropsy was performed on animals at the time of sacrifice. No macroscopic changes that could be attributed to the injection of MVAnef were detected. There were no differences in spleen weight between the groups of animals (data not shown). During the course of the experiment, two animals immunized with MVAnef died (one in group A and one in group B, see Figure 1 for immunization protocol and number of animals/group). One died as a result of a misplaced i.p. injection and the other from an unknown cause. The latter animal could not be subjected to necropsy due to post-mortem damage inflicted by the cage mates.

#### Histopathology

Minor incidental changes were noted in some of the analyzed organs; they were considered to be related to the background pathology seen in mice of this strain and age (Table 1). Minimal to slight lymphoid cell accumulation was noted in lungs of both MVA immunized animals and control animals, possibly an effect of the inhalation of isoflurane prior to sacrifice. No malignancies were noted in any of the animals. Two MVAnef injected animals had increased extra-medullary hematopoiesis of the spleen. A variation of lymphoid cell accumulation or minimal to slight inflammation occurred without indications of any malignancies or other serious pathological changes.

**Table 1:** Individual microscopic findings

Group:Animal	Lung	Liver	Spleen	Lymph Node	Kidney	Ovaries
MVAx3:1	0	0	0	0	0	nd
MVAx3:2	Congestion 3	0	0	nd	0	0
MVAx3:3	0	0	0	nd	0	nd
MVAx3:4	0	0	0	0	0	0
MVAx3:5	0	MiFo inflammation 2	0	0	Unilateral Chro pyelonephritis 1	0
MVAx3:6	Lymphoid cell acc. 2	MiFo inflammation 1	0	0	0	0
MVAx3:7	Lymphoid cell acc. 2	0	0	0	0	0
MVAx3:8	0	Peri. inflammation 2	Incr. extramedullary hematopoiesis 2	nd	0	0
MVAx3:9	0	0	0	nd	0	Unilateral cyst
MVAx3:10	Lymphoid cell acc. 1	Peri. inflammation 1	Incr. extramedullary hematopoiesis 1 Hemosiderosis 1	0	0	nd
PBSx2+MVAx1:1	Lymphoid cell acc. 1	MiFo inflammation 1	0	nd	0	0
PBSx2+MVAx1:2	0	0	0	nd	0	nd
PBSx2+MVAx1:3	0	0	0	nd	0	0
PBSx2+MVAx1:4	0	0	0	0	0	0
PBSx3:1	0	MiFo inflammation 2	Lymphoid hyperplasia 1	0	0	0
PBSx3:2	Lymphoid cell acc. 1	0	0	nd	0	0
PBSx3:3	0	0	0	nd	0	0
PBSx3:4	0	0	0	nd	0	nd
PBSx3:5	0	0	0	0	0	0
PBSx3:6	0	0	0	0	0	0

Abbreviations: acc = accumulation, MiFo = Microfocal, Incr = Increased, Peri = Periportal, Chro = Chronic, nd = not done

Grading of changes: 0 = No changes, 1 = Minimal changes, 2 = Slight changes, 3 = moderate changes, 4 = Marked changes, 5 = Severe changes,

#### Hematology

The bone marrow of all animals displayed normal cellularity (Table 2). Megakaryocytes were found in normal numbers and were of normal size. Staining for iron was positive in all bone marrow samples. The blood of all animals showed a moderate neutropenia, evaluated as a slight decrease of segmented granulocytes, while lymphocytes as well as monocytes were found in normal numbers. There were no immature cells in the peripheral circulation and no pathological proliferation of blood cells was noted. The red cells of animals in all groups, both vaccinated and controls, showed a slight to moderate degree of anisocytosis, poikilocytosis and polychromasia. No nucleated red cells could be found but many cells contained a Howell-Jolly body. Platelets were found in normal or slightly increased numbers. Despite the occurrence of moderate neutropenia in all animals, including the control animals that received intraperitoneal injections of PBS, no animal showed any toxic effect leading to a decreased cellularity of the bone marrow. The findings in immunized animals could not be distinguished from non-immunized animals and were attributed to background histology of animals of this strain and age.

**Table 2:** Individual hematopathology findings

	Blood Smears								Bone marrow		
Group:Animal	Segm	Ly/Mo	NRC	A	PC	PCR	HJB	Plt	Cellularity	Megakaryocytes	Iron contents

MVAx3:1	decr	nr	-	++	+	++	+	nr	100%	nr	+
MVAx3:2	decr	nr	-	+	+	+	+	nr	100%	nr	+
MVAx3:3	decr	nr	-	+	+	+	+	nr	100%	nr	+
MVAx3:4	decr	nr	-	+	+	++	++	nr	100%	nr	+
MVAx3:5	decr	nr	-	+	+	+	+	incr	100%	nr	+
MVAx3:6	decr	nr	-	+	+	+	+	nr	100%	nr	+
MVAx3:7	decr	nr	-	(+)	-	-	+	incr	100%	nr	+
MVAx3:8	decr	nr	-	+	+	+	+	nr	100%	nr	+
MVAx3:9	decr	nr	-	+	+	+	+	nr	100%	nr	+
MVAx3:10	decr	nr	-	+	+	+	+	nr	100%	nr	+
PBSx2+MVAx1:1	decr	nr	-	+	+	+	+	nr	100%	nr	+
PBSx2+MVAx1:2	decr	nr	-	+	+	+	+	incr	100%	nr	+
PBSx2+MVAx1:3	decr	nr	-	+	+	+	+	nr	100%	nr	+
PBSx2+MVAx1:4	decr	nr	-	+	+	+	+	incr	100%	nr	+
PBSx3:1	decr	nr	-	+	+	+	+	incr	100%	nr	+
PBSx3:2	decr	nr	-	+	+	+	+	incr	100%	nr	+
PBSx3:3	decr	nr	-	+	+	+	+	incr	100%	nr	+
PBSx3:4	decr	nr	-	-	-	(+)	(+)	incr	100%	nr	+
PBSx3:5	decr	nr	-	-	-	(+)	(+)	incr	100%	nr	+
PBSx3:6	decr	nr	-	-	-	(+)	(+)	incr	100%	nr	+

Abbreviations: Segm = Segmented granulocytes, Ly/Mo = Lymphocytes/monocytes, NRC = Nucleated red cells, A = Anisocytosis, PC = Poikilocytosis, PCR = Polychromasia, HJB = Howell Jolly Bodies, Plt = Platelets, nr = non remarkable, incr = increased, decr = decreased

### Vaccine-induced HIV-1-specific immune responses

#### MVA boosts a DNA primed humoral Nef response

**Figure 2:**
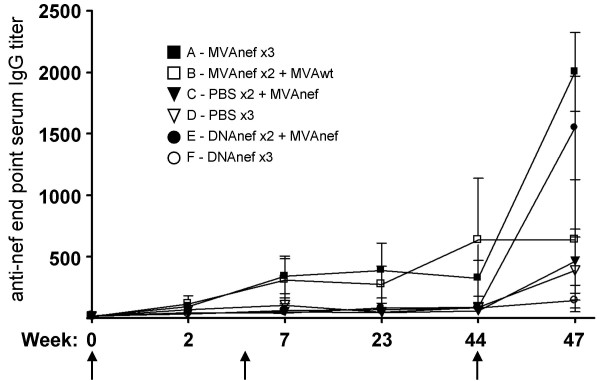
Humoral responses against nef. End point anti-Nef ELISA titers in sera from 5 different time points. Arrows indicate time of immunization (w 0, 5 and 44). Error bars show standard deviations of all animals in each group.

Animals primed with either DNAnef or MVAnef and subsequently boosted with MVAnef (groups E and A, respectively) displayed significantly stronger humoral responses against Nef than animals in the other groups (Figure 2). Interestingly, three injections of plasmid-encoded Nef did not induce Nef-specific antibodies. However, a single boost with MVAnef served to enhance the antibody response following a DNA prime to the same levels as after three injections with MVAnef.

#### DNA and MVA induce Nef-specific cellular responses

**Figure 3:**
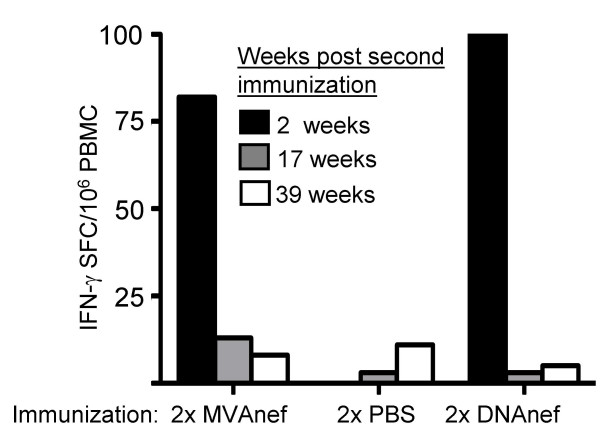
IFN-γ ELISpot secretion by PBMC following stimulation with peptides covering Nef. Results from 3 time-points following the two initial immunizations but prior to the boost at week 44.

Cellular immune responses in peripheral blood were assessed at three time points following the two initial injections (Figure 3). Significant cellular responses to Nef were detected two weeks after the second immunization. Animals immunized with either DNAnef or MVAnef displayed a Nef-specific cellular response as measured by IFN-γ production after stimulation of PBMC with overlapping peptides representing the full Nef protein. This initial response declined over time and was undetectable at 17 weeks after the second immunization.

#### Similar levels of cellular responses after MVA and DNA late boost immunization

**Figure 4:**
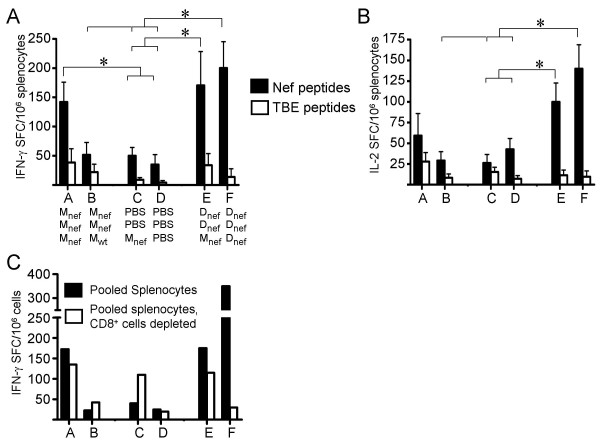
Nef responses as measured by ELISpot. (**A**) IFN-γ and (**B**) IL-2 secretion by splenocytes stimulated with either a pool of overlapping peptides covering Nef or a pool of control peptides (Tick Borne Encephalitis virus, TBE). * indicates a significant difference (p < 0.05). Error bars show standard deviations of all animals in each group. (**C**) IFN-γ secretion by pooled splenocytes from all animals in each group (black bars) or by pooled splenocytes depleted of CD8 + cells (white bars). M = MVA, D = DNA.

After the late boost (at week 44, corresponding to 39 weeks after the second immunization, see Figure 1), the most robust anti-Nef responses in splenocytes were seen in animals primed with DNAnef and boosted either with MVAnef (group E; DNAnef followed by MVAnef) or with an additional injection of DNAnef (group F; DNAnef followed by DNAnef) (Figure 4a). Animals in these two groups responded with comparable levels of IFN-γ production following stimulation of splenocytes with peptides overlapping Nef. Furthermore, the levels of IFN-γ responses by spleen cells displayed by these animals were similar to those in the animals immunized three times with MVAnef (group A; MVAnef followed by MVAnef). Nef-specific responses in animals primed twice with MVAnef and boosted with wild-type MVA (group B; MVAnef followed by MVAwt) were weak. Here, a third injection of MVAnef (group A) was needed to induce detectable cellular immune responses to Nef (Figure 4a). Injection of MVAnef once (group C; PBS followed by MVAnef) did not result in measurable immune responses (Figure 4a). Judging from IL-2 measurements (Figure 4b), the strongest responses were found in animals primed with DNAnef and boosted either with an additional injection of DNAnef (group F; DNAnef followed by DNAnef) or with MVAnef (group E; DNAnef followed by MVAnef). These results emphasize previous findings that plasmid DNA followed by a viral vector boost might be superior to a repeated immunization with the viral vector [26]. Importantly, we also show that DNA can be used as a late boost of DNA primed immune responses.

#### Plasmid-encoded Nef induces CD8^+ ^responses, whereas Nef encoded by MVA induces CD4^+ ^responses

The depletion of CD8^+ ^T cells from splenocytes followed by stimulation with Nef peptides showed that only animals immunized repeatedly with DNA (group F; DNAnef followed by DNAnef) developed a clear CD8^+ ^dependent IFN-γ response (Figure 4c). The responses in other groups were largely unaffected by the depletion of CD8^+ ^cells, indicating that the major part of the effector cells were of CD4^+ ^origin. A single injection of MVAnef (group C; PBS followed by MVAnef) resulted in a moderate CD4^+ ^response after stimulation with Nef peptides (Figure 4c). Taken together, these results indicate that MVAnef induces a long-lived CD4^+ ^T cell memory response, whereas repeated DNAnef immunization gives a long-lived CD8^+ ^response.

Intracellular cytokine staining of cryo-preserved splenocytes showed that MVAnef induced a potent response in terms of Nef-specific IFN-γ and TNF-α production by CD4^+ ^T cells (data not shown). This correlates well with the findings in the ELISpot assay of the CD4^+ ^responses. Due to high background and poor viability of the cryopreserved CD8 T cells, the intracellular cytokine staining of these cells gave no further information.

#### Vaccinia-specific immune responses

**Figure 5:**
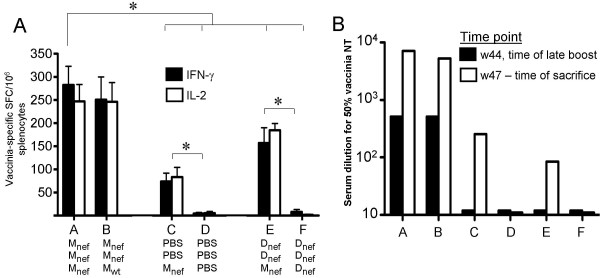
Reactivity against the vaccinia vector. **A**) IFN-γ and IL-2 secretion by splenocytes isolated 3 weeks following the late boost and stimulated with inactivated vaccinia. * indicates a significant difference (p < 0.05), M = MVA, D = DNA. Error bars show standard deviations of all animals in each group. **B**) End-point serum dilution for a 50% *in vitro *neutralization of vaccinia virus at the time of the late boost (black bars) and at the time of sacrifice, 3 weeks after the late boost (white bars).

The strongest cellular reactivity of splenocytes stimulated with inactivated vaccinia was observed in animals immunized three times with MVA (groups A and B, Figure 5a). Analogous to the cellular responses, the strongest vaccinia-neutralizing antibody responses were detected in the animals immunized three times with MVA (groups A and B, Figure 5b). These animals already had high titers of neutralizing antibodies two weeks after the two initial injections (titers of 512 for 50% neutralization of vaccinia). These responses persisted for the 39 weeks until the late boost (Figure 5b). Sera collected after the third injection from the animals immunized repeatedly with MVA (groups A and B) could be diluted several thousand times and still display more than 50% neutralization of vaccinia *in vitro *(Figure 5b). Animals immunized only once with MVA had antibody titers of 256 against vaccinia.

## Discussion

The inability of plasmid-based vaccines to induce long-lasting immune responses in humans has resulted in the development of alternative strategies for delivering genetic immunogens. The concept of heterologous prime/boost immunization, where two or more vaccine modalities are combined, is now rather well established in experimental vaccinology [27,28] and has also been evaluated in clinical trials [29,30]. One of the most promising prime/boost strategies is the combination of naked DNA with viral vectors. Several attenuated and modified viruses, including species within the families of Pox, Adeno and Alphaviruses, are being explored both as stand-alone vaccine vectors and as components of prime boost vaccine regimens.

We have used two constructs, plasmid DNA and Modified Vaccina virus Ankara (MVA), for delivering the HIV-1 nef gene. We assessed the long-term toxicology and immune responses following immunization with either vector alone or in a DNA prime MVA boost regimen. Both DNAnef and MVAnef have previously been shown to induce novel Nef-specific immune responses in HIV-1-infected humans [9,10]. During the clinical evaluation of the MVAnef construct in HIV-1 infected persons, several parameters were assessed to ensure the safety of the construct. The vaccinees were monitored for standard hematologic and laboratory safety parameters and immunization with MVAnef was concluded to be safe [10]. However, the ability of Nef to cause transformation in certain cell types *in vitro *[17-20] prompted us to perform an even more extensive toxicological evaluation of the vaccine, using a model permitting histopathological analysis of the major organs as well as analysis of the blood and bone marrow for the occurrence of tumors and other abnormalities. These types of extensive analysis are difficult to perform in a clinical trial since they require multiple organ biopsies.

We primed BALB/c mice with two injections of MVAnef or DNAnef and thirty-nine weeks later the animals received either a homologous or a heterologous boost. Three weeks after the boost, the animals were sacrificed and organs and blood were collected for toxicological as well as immunological analysis. The toxicological evaluation led to the conclusion that immunization of the mice with MVAnef did not cause pathological changes in the investigated organs or in the blood. Some abnormalities were noted, including accumulation of white blood cells in the lungs, a moderate decrease of segmented granulocytes in the blood and a slight to moderate degree of anisocytosis (abnormal size of the erythrocytes), poikilocytosis (irregularly shaped erythrocytes) and polychromasia of red blood cells. These observations were made in both immunized and control animals and were assigned to normal age-related pathology, not to effects of the vaccination. The absence of tumors and other adverse effects caused by the vaccine one year after the immunization is encouraging, since the animals were immunized with a massive dose of vaccine in relation to their body weight. We therefore conclude that immunizing with MVAnef does not induce any long-term toxicity.

One issue when using MVA as a vehicle for gene delivery is the anti-vector immunity that inevitably will be induced after immunization. Anti-vector immunity may neutralize the viral vector in subsequent vaccinations, possibly leading to a reduced capacity to boost the immune responses directed against the antigen expressed by the vector [31]. Strong cellular reactivity as well as high titers of vaccinia neutralizing antibodies were detected in animals immunized repeatedly with MVA (Figure 5). However, the immune responses against Nef could clearly be boosted by a third injection of MVAnef despite potent anti-vector reactivity (Figure 5b). The ability to boost immune responses despite the presence of neutralizing antibodies directed against the viral vector has been observed by others [32] and is of importance for clinical vaccine regimens where multiple MVA injections are given. The matter of anti-MVA reactivity is also of interest since the virus has been proposed as a candidate smallpox vaccine and there are results indicating that MVA is a potent inducer of immunity to smallpox [33,34]. The immunity to smallpox induced by the MVA would of course be an additional beneficial effect of using this particular viral vector for delivering other vaccine antigens.

The humoral and cellular immune responses against Nef were monitored during the forty weeks between the second and third immunizations. The two initial MVAnef immunizations induced prominent and stable long-lasting humoral responses against Nef. These results demonstrate the potential of MVA to induce a long-lasting B-cell memory to the vectored antigen, which is highly desirable for a vaccine vector. Intriguingly, when the animals primed with DNAnef were boosted with MVAnef, the humoral response to Nef increased to levels similar to those seen in the animals immunized repeatedly with MVAnef. This increase occurred despite undetectable antibody levels following the two priming DNAnef injections, demonstrating the capacity of plasmid-encoded Nef to prime humoral immune responses.

Interestingly, no significant difference in Nef-specific cellular responses could be detected when comparing the animals immunized repeatedly with either MVAnef or DNAnef or the prime/boost combination of the two vectors. Importantly, as discussed above for the Nef-specific humoral responses, the nef-specific cellular responses could also be readily boosted (Figure 4) despite the presence of anti-vector immunity (Figure 5).

The Nef-specific cellular responses that were detected two weeks after the second immunization, with either DNAnef or MVAnef, decreased over time (Figure 3) but were readily boosted forty weeks later (Figure 4), demonstrating the induction of a long-lasting cellular Nef-specific immunological memory. Importantly, a late boost with DNAnef could significantly enhance immune responses induced with the same DNA construct. These results suggest that the same plasmid vaccine can be used for late boosting and not only, as is common today, for priming of an immune response. This finding could have a great impact on upcoming DNA vaccine trials, as there are several advantages of naked DNA over viral vectors, including the lack of preexisting immunity to the plasmid vector DNA.

Ex-vivo depletion of CD8^+ ^cells from splenocytes revealed that only animals immunized with plasmid DNAnef three times over one year displayed a prominent CD8^+ ^dependent response. In contrast, animals immunized with MVAnef primarily developed CD4^+ ^responses to nef, a finding that was further confirmed by intracellular staining of cryo-preserved splenocytes. These findings are analogous to observations in human trials [10]. Interestingly, animals primed with plasmid DNA and subsequently boosted with MVA also predominantly exhibited CD4^+ ^and humoral responses, suggesting that MVAnef primarily induce a CD4^+ ^T cell memory, while repeated DNA immunization instead amplify the CD8 T cell response.

## Conclusion

To conclude, this study shows i) that the nef gene vectored by MVA does not induce malignancies or other adverse effects in mice and may safely be used as a vaccine in humans; ii) that a DNAnef prime followed by an MVAnef boost induced a strong and robust CD4^+ ^T cellular response balanced with high titers of Nef-specific antibodies; iii) that immunizing repeatedly with DNAnef induced a strong CD8^+ ^T cellular response, while low or no humoral or CD4^+ ^T cell responses could be detected; and iv) that it is possible to boost the Nef-specific responses using MVAnef despite the presence of neutralizing anti-vector antibodies.

Our findings have important implications for human immunizations since we demonstrate the possibility to induce a long-lasting immune memory, which can be readily boosted by an additional injection of DNA or recombinant MVA vaccine. Moreover, we demonstrate that it is possible to focus the immune response to either a CD4+ or CD8+ response by selecting the appropriate boosting vector.

## Methods

### Immunizations

The immunogens used were the plasmid DNAnef and MVAnef, both encoding wild type HIV-1_LAI _Nef. Both DNAnef and MVAnef have been described elsewhere [10,35] and shown to induce Nef-specific immune responses in humans [9,10]. Five-week-old female BALB/c mice (Charles River, Germany) were immunized at week 0 and week 5 with either 100 μg of DNAnef intramuscularly (10 mice, 50 μg DNA/hind leg, 2 mg DNA/ml saline), 10^8 ^pfu of MVAnef intraperitoneally (15 mice, 10^9 ^pfu/ml) or 100 μl saline intraperitoneally. Forty-four weeks following the initial immunization, each group of animals was split into two and boosted according to figure 1. Blood was drawn from the animals at weeks 2, 7, 23 and 44. Three weeks after the last injection the animals were sacrificed and organs and blood were harvested.

### Toxicology and histopathology

Animals were anaesthetized with isofluran and sacrificed by cervical dislocation. A licensed veterinarian performed necropsy at the time of sacrifice and the subsequent toxicological examination was performed by the GLP accredited laboratory at BioVet AB (Sollentuna, Sweden). The spleen from each animal was weighed and the following organs were preserved in 4% neutral buffered formaldehyde solution for subsequent examination: lungs, liver, spleen, inguinal lymph nodes, kidneys, ovaries and bone marrow. The listed organs were embedded in Histowax (Histolab Products AB, Sweden) and, after dehydration, sectioned at 4–6 μm, stained with hematoxylin and eosin and subsequently examined microscopically for pathological changes. Blood smears from each animal were prepared from peripheral blood and air-dried at the time of necropsy. The smears were fixed in methanol, stained according to the method of May-Grünewald-Giemsa and examined microscopically.

### ELISA

ELISA plates (Nunc MaxisorpOdense, Denmark) were coated with recombinant Nef (1.5 μg/ml) overnight at room temperature. The plates were blocked for 1 hour with 5% fat-free milk in PBS. Serum from each animal was diluted in 2.5 % fat-free milk in PBS and added to the wells. After 12 hours, reactive antibodies were detected with goat anti-mouse IgG antibodies conjugated to HRP (DAKO PO449, Denmark) diluted 1/3500 in 1.25 % fat-free milk. The plates were then developed for 30 min by adding O-phenylene diamine (Sigma, Sweden). The color reaction was stopped with 2.5 M H_2_SO_4 _and the optical density (OD) was read at 490 nm. The sera were considered positive for anti-Nef antibodies if the OD exceeded the mean value for negative samples (pre-immunization bleedings) plus 3 standard deviations.

### Neutralization of Vaccinia

Sera from animals in each group were pooled and inactivated at 56°C for 30 minutes and subsequently diluted. Diluted sera and vaccinia virus (Virus strain Elstree, Bernabiotech, Switzerland) of a final concentration of 167 pfu/ml were mixed in EMEM (2% FCS, Sigma, Sweden) and incubated for 90 minutes at 37°C. Virus and serum mixtures were then added in triplicates to fully confluent green monkey kidney (GMK) cells placed in 48 well plates. After incubation for 1 hour at 37°C, the serum and virus mixture was removed and replaced with 0.5 ml fresh EMEM (2% FCS). Cells were subsequently placed in 5% CO_2 _at 37°C. Forty hours later, the cells were stained and fixed by addition of 50 μl crystal violet diluted in 12% paraform aldehyde. Thirty minutes later, medium was removed and the cells were allowed to air-dry. The number of plaques formed in each well was counted in a light microscope. The neutralization capacities of sera from immunized animals were determined by comparing neutralizing activities of sera from the non-immunized animals as well as sera from blood drawn prior to the immunizations.

### ELISpot

The extraction and ficoll-paque (Amersham Biosciences Europe GmbH, Uppsala, Sweden) purification of splenocytes and PBMC were carried out as previously described [36]. For the depletion of CD8^+ ^T cells, Dynabeads (Dynal Biotechtech, Oslo, Norway) were used according to the manufacturer's instructions. The efficiency of CD8^+ ^cell depletion was confirmed by flow cytometry. Total and CD8^+ ^depleted splenocytes (10^6^) were stained for 30 min at 4°C with FITC conjugated anti CD4 antibodies and with PerCP conjugated anti-CD8a antibodies (BD Pharmingen). On average, 97% ± 1.4% of the CD8^+ ^cells were removed. Splenocytes from individual animals or pooled splenocytes from all animals in each group, before and after CD8^+ ^T cell depletion, were suspended in RPMI 1640 (Sigma, Sweden) supplemented with penicillin/streptomycin (PEST, Invitrogen Corporation, Carlsbad, CA, USA) and 10% fetal calf serum (FCS, Sigma, Sweden) and were distributed in anti-Interferon-γ (IFN-γ) (Mabtech, Nacka, Sweden) antibody coated 96-well polyvinylidene fluoride (PVDF) bottomed plates (MAIPN 4510, Millipore Corporation, Bedford, MA, USA). Splenocytes, 2 × 10^5^/well, were stimulated either with 15-mer peptides (overlapping by 10 aa, 5 μg/peptide/ml) covering Nef or with heat-inactivated vaccinia (95°C, 30 min) virus, 5 × 10^6 ^pfu/well. As negative controls, a peptide library consisting of 18 peptides derived from tick-borne encephalitis virus (5 μg/ml/peptide) or medium alone was used. Concanavalin A (1 μg/well, Sigma, Sweden) was used to confirm cell viability. The plates were then developed as described by the manufacturer and read in an ELISpot reader (AID, Germany). Results are given as cytokine-producing spot-forming cells (SFC) per million plated cells and responding animals were defined as having above 50 SFC per million cells and twice the reactivity of unstimulated cells from the same animal.

### Intracellular cytokine staining

Frozen splenocytes from vaccinated mice were thawed and incubated overnight at 37°C. The next day 12 × 10^6 ^cells of each mouse spleen were pooled to obtain representative pools for each vaccination group. Splenocytes were stimulated with either a peptide pool covering the whole Nef protein by 15-mer peptides (overlapping by 10 aa) or a control peptide at 1 μg/peptide/ml in the presence of 1 μg/ml Brefeldin A (Sigma) for 5 h. The subsequent staining of cells was carried out according to previously described procedures [37]. Briefly, the cells were incubated for 20 min with ethidium monazide (Molecular Probes) for live/dead discrimination and anti-Mouse-Fc-Ab (Pharmingen) to avoid unspecific binding of surface marker antibodies for 20 min and washed three times. Surface markers were stained with PE conjugated anti-CD8α, PerCP conjugated anti-CD4 and APC conjugated anti-CD62L (all Pharmingen) and washed again three times. Intracellular cytokine staining for IFN-γ and TNF-α production was performed by using the Cytofix/Cytoperm kit (Pharmingen) according to the manufacturer's recommendations. Data were acquired on a FACScan (Becton Dickinson) and further analyzed with FlowJo (Tree Star) software.

### Statistical analysis

Statistical analyses were performed using the SPSS program, version 10.1.0 for Windows. The criterion for statistical significance was p ≤ 0.05. Since most of the data were not normally distributed, the non-parametric Kruskal-Wallis test and the Mann-Whitney U test were used. Following a statistically significant Kruskal-Wallis test, the Mann-Whitney U test was used for pairwise post hoc comparison.

## Authors' contributions

ABråve, LG, KH, ER, ABoberg and GE all helped with the ELISpot, the ELISA and the vaccinia neutralization and interpreted the results. SR performed and interpreted the histopathological examinations. GG, WK, AC, and ID developed the MVA construct and performed the intracellular cytokine staining, besides helping to draft the manuscript. ABråve, JH, BW and VE all participated in planning the study, as well as in drafting and finalizing the manuscript. All authors read and approved the final manuscript.
